# A Plant-Based Dietary Intervention Improves Beta-Cell Function and Insulin Resistance in Overweight Adults: A 16-Week Randomized Clinical Trial

**DOI:** 10.3390/nu10020189

**Published:** 2018-02-09

**Authors:** Hana Kahleova, Andrea Tura, Martin Hill, Richard Holubkov, Neal D. Barnard

**Affiliations:** 1 Physicians Committee for Responsible Medicine, Washington, DC 20016, USA; nbarnard@pcrm.org; 2 Metabolic Unit, CNR Institute of Neuroscience, 35127 Padua, Italy; andrea.tura@cnr.it; 3 Institute of Endocrinology, 11394 Prague, Czech Republic; mhill@endo.cz; 4 School of Medicine, University of Utah, Salt Lake City, UT 84132, USA; richard.holubkov@hsc.utah.edu; 5 Adjunct Faculty, George Washington University School of Medicine and Health Sciences, Washington, DC 20016, USA

**Keywords:** beta-cell function, diet, nutrition, diabetes, vegan

## Abstract

The aim of this study was to test the effect of a plant-based dietary intervention on beta-cell function in overweight adults with no history of diabetes. Participants (*n* = 75) were randomized to follow a low-fat plant-based diet (*n* = 38) or to make no diet changes (*n* = 37) for 16 weeks. At baseline and 16 weeks, beta-cell function was quantified with a mathematical model. Using a standard meal test, insulin secretory rate was calculated by C-peptide deconvolution. The Homeostasis Model Assessment (HOMA-IR) index was used to assess insulin resistance while fasting. A marked increase in meal-stimulated insulin secretion was observed in the intervention group compared with controls (interaction between group and time, Gxt, *p* < 0.001). HOMA-IR index fell significantly (*p* < 0.001) in the intervention group (treatment effect −1.0 (95% CI, −1.2 to −0.8); Gxt, *p* = 0.004). Changes in HOMA-IR correlated positively with changes in body mass index (BMI) and visceral fat volume (*r* = 0.34; *p* = 0.009 and *r* = 0.42; *p* = 0.001, respectively). The latter remained significant after adjustment for changes in BMI (*r* = 0.41; *p* = 0.002). Changes in glucose-induced insulin secretion correlated negatively with BMI changes (*r* = −0.25; *p* = 0.04), but not with changes in visceral fat. Beta-cell function and insulin sensitivity were significantly improved through a low-fat plant-based diet in overweight adults.

## 1. Introduction

Impairment of pancreatic beta-cell function, typically preceded by insulin resistance in muscle and liver cells, is a key factor in type 2 diabetes [1]. Improvements in beta-cell function, however, are not typically a goal of diabetes treatment. Beta-cell failure reflects a relative loss of beta-cell mass due to apoptosis [2], as well as selective loss of sensitivity to glucose, along with the loss of first-phase insulin secretion [3]. Limited data suggest that beta-cell function and beta-cell mass may be influenced by diet [4,5,6] and physical activity [4].

Methods used for quantifying beta-cell function include the homeostasis model assessment (HOMA), oral glucose tolerance tests, intravenous glucose tolerance tests, hyperglycemic clamp, and standard meal tolerance tests. No single test allows beta-cell function to be assessed with accuracy and specificity comparable to those of insulin sensitivity. Therefore, mathematical modeling is necessary to interpret parameters of beta-cell function in all of the mentioned methods. The use of oral glucose tolerance tests or standard meal tests has been preferred due to simplicity but also for physiological significance, as they include the gastrointestinal incretin effect that follows oral nutrient ingestion [7,8]. 

Several prospective studies have demonstrated that diabetes prevalence is 46–74% lower among individuals following plant-based diets, compared with the general (non-vegetarian) population, even after adjustment for differences in BMI [5,6,9]. In addition, a vegan diet has been shown to improve glycemic control in type 2 diabetes compared with a more conventional hypocaloric, carbohydrate-controlled diet [10,11]. These studies raise the question as to whether such a diet intervention could improve beta-cell function and insulin sensitivity. The aim of this study was to test the effect of a dietary intervention on beta-cell function and insulin resistance in overweight adults with no history of diabetes.

## 2. Materials and Methods

### 2.1. Study Design and Eligibility

The study was conducted between October 2016 and June 2017 in Washington, DC, using a single-center, randomized, open parallel study. Men and women, aged 25 to 75 years, with a body-mass index between 28 and 40 kg/m^2^, were enrolled. Exclusion criteria were history of diabetes, smoking, alcohol or drug abuse, pregnancy or lactation, and current use of a vegan diet. The study protocol was approved by the Chesapeake Institutional Review Board on 12 October 2016. The protocol identification number is Pro00018983. All participants gave written informed consent. Trial Registration: ClinicalTrials.gov number, NCT02939638.

### 2.2. Randomization and Study Groups

The participants were randomly assigned in a 1:1 ratio to an intervention group or a control group. Participants were examined at baseline and at 16 weeks.

The intervention group was asked to follow a low-fat vegan diet (~75% of energy from carbohydrates, 15% protein, and 10% fat) consisting of vegetables, grains, legumes, and fruits. Participants were instructed to avoid animal products and added fats. Daily fat intake was 20–30 g to ensure adequate intake of essential fatty acids. No meals were provided. Vitamin B_12_ was supplemented (500 µg/day). The control group was asked to make no diet changes. Alcoholic beverages were limited to one per day for women and two per day for men in both groups.

To monitor adherence, a 3-day dietary record was completed by each participant at baseline and at 16 weeks. Dietary intake data were collected and analyzed by a registered dietician, using Nutrition Data System for Research version 2016, developed by the Nutrition Coordinating Center (NCC), University of Minnesota, Minneapolis, MN, USA [12]. In addition, study dietitians made unannounced telephone calls to assess participants’ dietary adherence. All study participants were asked not to alter their exercise habits, and to continue their preexisting medication regimens for the duration of the study, except as modified by their personal physicians. Physical activity was assessed by the International Physical Activity Questionnaire (IPAQ) [13].

### 2.3. Outcomes

All measurements were performed at baseline and 16 weeks on an outpatient basis, after a 10–12 h overnight water-only fast. Height and weight were measured using a stadiometer and a periodically calibrated scale accurate to 0.1 kg, respectively. Body composition was assessed using a DXA scan (iDXA; GE Healthcare, Chicago, IL, USA).

Insulin secretion was assessed after stimulation with a liquid breakfast (Boost Plus, Nestle, Vevey, Switzerland; 720 kcal, 34% of energy from fat, 16% protein, 50% carbohydrate). Plasma concentrations of glucose, immunoreactive insulin, and C-peptide were measured at 0, 30, 60, 120, and 180 min. Serum glucose was analyzed using the Hexokinase UV endopoint method (Roche, Basel, Switzerland). Plasma immunoreactive insulin and C-peptide concentrations were determined using insulin and C-peptide electro-chemiluminescence immunoassay (ECLIA) kits (Roche, Basel, Switzerland). HbA1c was measured by turbidimetric inhibition immunoassay (Roche, Basel, Switzerland). Plasma lipids concentrations were measured by enzymatic colorimetric methods (Roche, Basel, Switzerland).

The primary outcome was beta-cell function assessed by a mathematical model [14,15,16], as follows: Insulin secretory rates were calculated from plasma C-peptide concentrations by deconvolution [14] and expressed per square meter of estimated body surface area. The dependence of insulin secretory rates on glucose concentrations was modeled separately for each participant and each study day.

Insulin secretion consists of two components. The first represents the static dependence of insulin secretion on glucose concentration and is characterized by a dose-response function. Its parameters are insulin secretion at 5 mmol/L glucose (fasting glucose level) and mean slope in the glucose range. The dose response is modulated by a potentiation factor, quantified as the ratio between 160–180 and 0–20 min values. The second component represents a dynamic dependence of insulin secretion on the rate of change of glucose and is determined by the rate sensitivity [15,16]. The model parameters (i.e., the parameters of the dose response, and the potentiation factor) were estimated from glucose and C-peptide concentration by regularized least squares [14,15,16]. Regularization involves the choice of smoothing factors that were selected to obtain glucose and C-peptide model residuals with standard deviations close to the expected measurement error (~1% for glucose and ~5% for C-peptide). C-peptide has been used for the calculation of insulin secretion, as it is secreted with insulin in an equimolar fashion, but does not undergo liver elimination. Therefore, plasma C-peptide provides a more accurate estimation of insulin secretion. Plasma insulin has been used for the assessment of insulin sensitivity, both in fasting and stimulated conditions. Estimation of the individual model parameters was performed by an investigator masked to group assignment.

The secondary outcome was insulin resistance calculated as Homeostasis Model Assessment (HOMA-IR) index [17]. In addition, oral glucose insulin sensitivity index was calculated as a measure of dynamic postprandial insulin sensitivity [18].

### 2.4. Statistical Analysis

The intention-to-treat analysis included all participants, regardless of their subsequent withdrawal or non-adherence to the prescribed diet [19]. A repeated measure ANOVA model was used to test the between-group differences from baseline to 16 weeks with between-subject and within-subject factors and interactions. Group, subject and time were included in the model. Interaction between group and time (Gxt) was calculated for each variable. For the dose-response analysis of insulin secretion as a function of plasma glucose concentrations, glucose concentration was added to the ANOVA model. Within each group, paired comparison *t*-tests were calculated to test whether the changes from baseline to 16 weeks were statistically significant. Pearson correlations were calculated for the relationship between changes in HOMA-IR and beta-cell function, and changes in BMI and volume of visceral fat.

## 3. Results

### 3.1. Characteristics of the Participants

**Figure 1:**
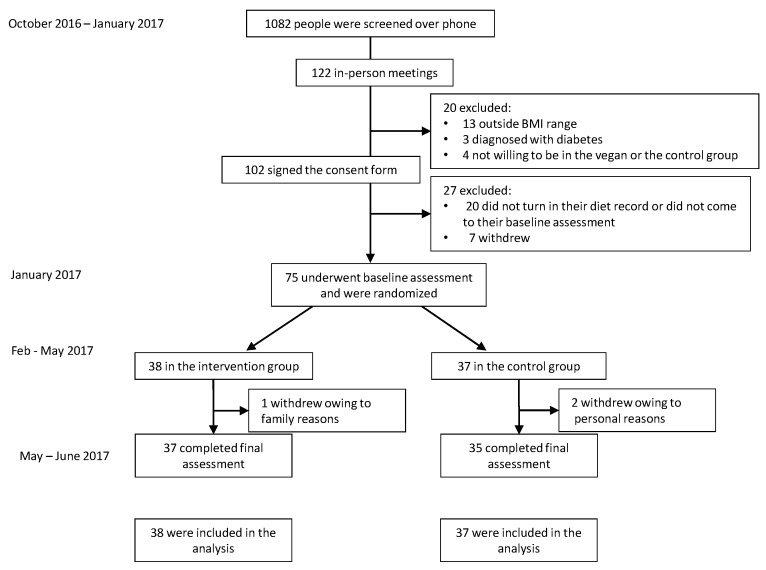
Enrollment of the participants and completion of the study.

Of 1082 people screened by telephone, 75 met the participation criteria and were randomly assigned to the intervention (*n* = 38) or control (*n* = 37) groups, and 96% of participants completed the study. Two participants dropped out of the control group and one from the intervention group, all for reasons not related to the diet (Figure 1). Demographic characteristics are listed in Table 1. Baseline physical activity, dietary intake, anthropometric and laboratory variables, parameters of insulin resistance and beta-cell function, as well as their changes in response to the intervention are shown in Table 2.

### 3.2. Physical Activity and Dietary Intake

Physical activity did not change substantially in either group. Both groups reduced reported energy intake (*p* < 0.001 for the intervention group, and *p* = 0.009 for controls), with no significant difference between groups (*p*-value for interaction between the factors group and time, Gxt, *p* = 0.69). Mean intake of carbohydrate, fat and protein did not change significantly in control participants, but there were significant reductions in their mean saturated fatty acid intake (*p* = 0.002) and the glycemic index of their diets (*p* = 0.03). The intervention group participants increased their mean intake of carbohydrate (*p* < 0.001) and fiber (*p* < 0.001), while decreasing consumption of total fat (*p* < 0.001), as well as saturated (*p* < 0.001), polyunsaturated (*p* < 0.001), and monounsaturated fatty acids (*p* < 0.001), cholesterol (*p* < 0.001), and protein (*p* < 0.001).

### 3.3. Body Mass Index and Body Composition

Body mass index decreased significantly only in the intervention group (Gxt, *p* < 0.001). While lean mass was reduced in both groups (Gxt, *p* = 0.002), fat mass, and particularly visceral fat volume, were reduced only in the intervention group (Gxt, *p* < 0.001; Gxt, *p* < 0.001, respectively).

### 3.4. Laboratory Variables

Significant reductions in total (*p* < 0.001; Gxt, *p* = 0.02), LDL- (*p* = 0.002; Gxt, *p* = 0.03), and HDL-cholesterol (*p* < 0.001; Gxt, *p* = 0.002), were observed in the intervention group. In addition, fasting plasma glucose (*p* = 0.002; Gxt, *p* < 0.001), insulin (*p* = 0.01; Gxt, *p* = 0.05), and C-peptide (*p* < 0.001; Gxt, *p* = 0.003) all fell in the intervention group. There were no significant changes in plasma lipid concentrations or markers of glycemic control in the control group.

### 3.5. Beta-Cell Function

**Figure 2:**
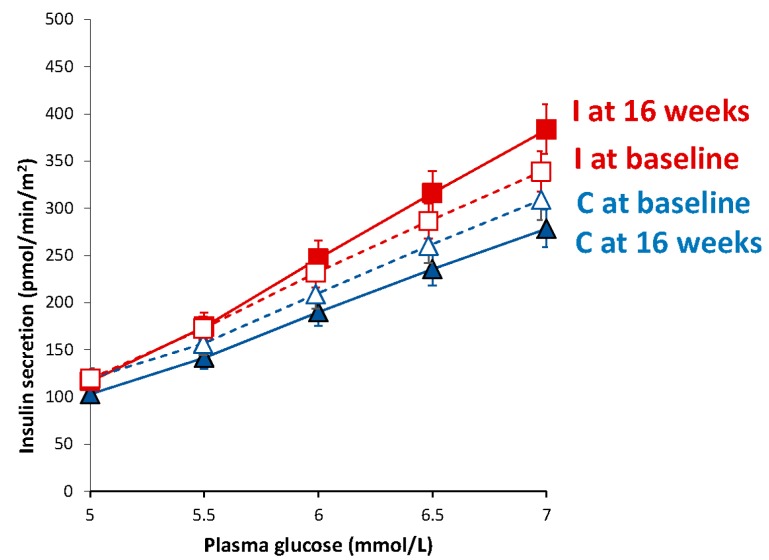
Dose-response insulin secretion in response to different plasma glucose levels. Triangles with the blue line represent the control group (empty triangles and a dashed line at baseline, and full triangles with a full line at 16 weeks), while squares with the red line show data from the intervention group (empty squares with a dashed line at baseline, and full squares with a full line at 16 weeks). Data are given as means with 95% CIs.

In the intervention group, we observed a decrease in basal insulin secretion (*p* = 0.006; treatment effect −54.2 (95% CI −86.5 to −21.9) pmol/min/m^2^; Gxt, *p* < 0.001). The intervention group also had increased beta-cell glucose sensitivity (*p* = 0.01), although the difference between groups did not reach statistical significance (treatment effect +65.5 (95% CI −74.4 to +205.4) pmol/min/m^2^/mM; Gxt, *p* = 0.13). Parameters of beta-cell function did not change significantly in controls, except for an increase in total insulin secretion (*p* = 0.008). A marked dose-response increase in insulin secretion as a function of plasma glucose concentrations (Figure 2) was observed in the intervention group compared with controls (Gxt, *p* < 0.001).

### 3.6. Insulin Resistance

HOMA-IR index fell significantly (*p* < 0.001) in the intervention group, but did not change significantly in controls (treatment effect −1.0 (95% CI, −1.2 to −0.8); Gxt, *p* = 0.004). No significant change in oral glucose insulin sensitivity was observed in either group.

### 3.7. The Effect of BMI and Adiposity

Changes in HOMA-IR correlated positively with changes in BMI and volume of visceral fat (*r* = 0.34; *p* = 0.009; and *r* = 0.42; *p* = 0.001); the latter remained significant even after adjustment for changes in BMI (*r* = 0.41; *p* = 0.002). Changes in beta-cell function, evidenced by glucose-induced insulin secretion, correlated negatively with changes in BMI (*r* = −0.25; *p* = 0.04), but not with changes in visceral fat. 

## 4. Discussion

In this randomized, controlled 16-week trial, beta-cell function and insulin resistance were altered by a dietary intervention. The dietary intervention elicited marked increases in meal-stimulated insulin secretion and beta-cell glucose sensitivity, along with decreased fasting insulin resistance and decreased fasting and postprandial plasma glucose concentrations, in individuals with no history of diabetes.

Previously, we [20] and others [4] demonstrated improvements in beta-cell function in individuals with type 2 diabetes with an energy-restricted diet. The current study demonstrates that, in individuals who are overweight but have no history of diabetes, a qualitative change in macronutrient composition with no limit on energy intake, elicits an improvement in beta-cell function and fasting insulin resistance.

In the control group, plasma glucose levels increased during the meal test, resulting in an increase in total insulin secretion. Therefore, beta-cell glucose sensitivity (which is essentially secretion “normalized” for plasma glucose levels) did not improve. This physiologic reaction of beta-cells to compensate for impaired glycemic control, together with insulin resistance, which had a tendency to increase in controls, are two main pathophysiologic mechanisms in development of beta-cell dysfunction, and eventually diabetes [1], with reduced beta-cell function playing the decisive role [3]. Conversely, the intervention group participants improved their metabolic condition; glucose sensitivity improved, as did fasting insulin sensitivity, with a decrease in fasting plasma glucose and mean glucose levels during the meal test.

Fasting insulin resistance, calculated as the HOMA-IR index, decreased (i.e., improved) in the intervention group, whereas no changes under dynamic postprandial conditions were observed in either group. As fasting insulin resistance (HOMA-IR) primarily reflects hepatic insulin resistance (whereas dynamic postprandial insulin sensitivity indices, such as oral glucose insulin sensitivity, capture mainly skeletal muscle metabolism [18], our results suggest a marked improvement in hepatic, rather than peripheral, insulin sensitivity. The decrease in insulin resistance was related to loss of visceral fat, independent of changes in BMI, while changes in glucose-induced insulin secretion were related to changes in BMI only. Our results are in accordance with a recent paper showing that metabolic crosstalk of fatty liver with pancreatic islets may contribute to obesity-related impairment of beta-cell glucose-sensitivity [21]. In this context, it seems plausible that a low-fat vegan diet in our study decreased hepatic insulin resistance and led to a subsequent improvement in beta-cell function. This is in line with the findings from the Finnish Diabetes Prevention Study, showing that the reduction in the risk of development of type 2 diabetes after a lifestyle intervention is related to improvement in insulin sensitivity, which has beneficial effects on preservation of beta-cell function [22,23]. However, we note that our study assessed the effect of the intervention overall and was not designed to differentiate the effects of the elimination of animal products, the reduction in overall fat intake, and the reduction in body weight that these changes elicit. Among lifestyle interventions associated with weight loss, plant-based diets are particularly effective [24,25,26].

The improvement in plasma lipid concentrations in response to a low-fat vegan diet is in accordance with previous studies, summarized in a recent meta-analysis, which showed that plant-based diets are associated with decreased total cholesterol, both LDL-, and HDL-cholesterol, but not with decreased triglycerides [27]. Improved blood lipids appear to play an important role in the cardio-metabolic benefits and reduced all-cause mortality observed with vegetarian and vegan diets [28].

Beta-cell function is improved by therapies that reduce body fat (such as diet and exercise, GLP-1 agonists, or bariatric surgery) or change fat distribution (such as thiazolidinediones) [29,30]. Vegetarian and vegan diets have been shown to be effective for weight loss and particularly for reduction in visceral fat and subfascial fat in muscle tissue [24,25,31,32], which is involved in glucose homeostasis [33]. 

Additional possible mechanisms for improved beta-cell function observed with the dietary intervention involve the incretin hormones, which are released from the gastrointestinal tract in response to nutrient ingestion to enhance meal-dependent insulin secretion from the pancreas [34]. Compromised incretin action plays a role in the development of beta-cell dysfunction and type 2 diabetes [35]. We showed previously that a plant-based diet improved incretin secretion [36]. Therefore, in the present study, it is conceivable that the intervention diet may have improved beta-cell function through enhanced incretin action. Other factors that have been hypothesized to be involved in improvement of beta-cell function include reduced lipotoxicity, glucotoxicity, oxidative stress, and inflammation; in theory, each of these can be influenced by diet [31,37]. A high-carbohydrate diet has been shown to improve insulin sensitivity and meal-stimulated insulin secretion in individuals with impaired fasting glucose [38]. Therefore, the substantially increased carbohydrate intake might have played an important role in the improvement of beta-cell function in our study. In addition, while dietary cholesterol has been shown to induce cellular and mitochondrial oxidative stress and lipid peroxidation, leading to beta-cell dysfunction, quercetin and other polyphenols have anti-apoptotic, antioxidant and anti-inflammatory properties and seem to improve ATP-linked mitochondrial energy metabolism, thereby preserving meal-stimulated insulin secretion and beta-cell function [39,40].

The strengths of the study include the randomized parallel design, in which all participants started simultaneously, allowing the investigators to rule out possible effects of seasonal fluctuations in the diet. The study duration was reasonably long, providing sufficient time for adaptation to the diet. We used physiological stimulation by a standard mixed meal, enabling us to study beta-cell function during a physiological perturbation. A unique feature of our study lies in the detailed assessment of beta-cell function, using mathematical modelling. The low attrition rate suggests that the intervention was acceptable and sustainable, in accordance with the findings of a previous long-term study [11]. Given that the participants were living at home and preparing their own meals or eating at restaurants, our results are applicable outside the research setting, in free-living conditions. The study also has important limitations. Dietary intake was calculated based on self-reported diet records, which have well-known limitations [41]. However, it is reassuring that the reported changes in nutrient intake were paralleled by weight loss and metabolic changes. Our participants were generally health-conscious individuals who were willing to make substantial changes to their diet. In this regard, they may not be representative of the general population, but may be representative of a clinical population seeking help for weight problems.

## 5. Conclusions

In conclusion, we have demonstrated that beta-cell function and fasting insulin sensitivity can be modified by a 16-week dietary intervention. Our study suggests the potential of a low-fat plant-based diet in diabetes prevention, addressing both core pathophysiologic mechanisms—insulin resistance and diminished beta-cell function—at the same time.

## Figures and Tables

**Table 1:** Baseline characteristics of the Study Population. Data are means ± SD, or number (%). *p*-values refer to *t*-tests for continuous variables and χ^2^ for categorical variables. The *p*-value calculated for ethnicity distribution is for the comparison between Hispanic vs. non-Hispanic categories.

Characteristic	Intervention Group (*n* = 38)	Control Group (*n* = 37)
Age (years)	52.6 ± 14.7	54.3 ± 9.9
Sex (number, %)		
Male	2 (5%)	6 (16%)
Female	36 (95%)	31 (84%)
Race, (number, %)		
White	15 (39%)	19 (51%)
Black	20 (53%)	14 (38%)
Asian, Pacific Islander	0	4 (11%)
American Indian, Eskimo, Aleut	2 (5%)	0
N/A—did not disclose	1 (3%)	0
Ethnicity, (number, %)		
Non-hispanic	33 (87%)	31 (84%)
Hispanic	3 (8%)	3 (8%)
N/A—did not disclose	2 (5%)	3 (8%)
Marital status		
Not married	22 (58%)	19 (51%)
Married	16 (42%)	17 (46%)
NA	0	1 (3%)
Education		
High school	0	0
College	17 (45%)	20 (54%)
Graduate degree	20 (53%)	17 (46%)
NA	1 (2%)	0
Occupation		
Service occupation	5 (13%)	1 (3%)
Technical, sales, administrative	15 (39%)	10 (27%)
Professional or managerial	10 (26%)	16 (43%)
Retired	4 (11%)	6 (16%)
Other	4 (11%)	4 (11%)
Medications		
Lipid-lowering therapy (%)	5 (13)	4 (11)
Antihypertensive therapy (%)	11 (29)	7 (19)
Thyroid medications (%)	6 (16)	3 (8)

**Table 2:** Changes in dietary intake, clinical and laboratory variables during the study. Data are means ± SD. Listed *p*-values are for interactions between group and time assessed by repeated measures ANOVA. * *p* < 0.05, ** *p* < 0.01 and *** *p* < 0.001 for within-group changes from baseline assessed by paired comparison *t*-tests.

	Control Group		Intervention Group		Treatment Effect	*p*-Value
	Baseline	Week 16	Baseline	Week 16		
**Total physical activity (METs)**	2642 (1476–3809)	2575 (1169–3980)	2207 (1444–2969)	2490 (1586–3395)	+351 (−1143 to +1846)	0.46
**Dietary intake**						
Caloric intake (kcal/day)	1923 (1627–2219)	1582 (1368–1795) **	1851 (1695–2007)	1450 (1249–1652) ***	−60 (−352 to +233)	0.69
Carbohydrates (% of daily energy)	45.5 (42.6–48.4)	46.6 (42.9–50.4)	46.1 (43.5–48.8)	69.6 (67.3–71.8) ***	+22.3 (+17.7 to +26.9)	<0.001
Fats (% of daily energy)	35.6 (32.3–38.9)	35.0 (31.5–38.4)	36.1 (34.0–38.1)	17.5 (15.5–19.4) ***	−17.9 (−22.3 to −13.6)	<0.001
Proteins (% of daily energy)	16.1 (15.0–17.1)	17.0 (15.5–18.5)	16.8 (15.4–18.2)	12.3 (11.3–13.3) ***	−5.4 (−7.8 to −3.0)	<0.001
Fiber intake (g/day)	25.2 (20.9–29.6)	23.5 (19.6–27.4)	24.2 (21.0–27.4)	37.8 (31.4–44.1) ***	+15.3 (+8.0 to +22.6)	<0.001
Cholesterol intake (mg/day)	290 (220–360)	212 (149–275)	264 (213–315)	6.5 (2.5–10.5) ***	−180 (−278 to −82)	<0.001
Saturated fatty acids (g/day)	25.5 (19.8–31.1)	17.9 (13.6–22.2) **	24.5 (21.2–27.7)	5.6 (4.6–6.7) ***	−11.2 (−16.5 to −5.9)	<0.001
Monounsaturated fatty acids (g/day)	30.5 (23.8–37.2)	24.9 (19.8–30.0)	28.9 (25.1–32.7)	9.5 (7.8–11.2) ***	−13.8 (−19.5 to −8.2)	<0.001
Polyunsaturated fatty acids (g/day)	20.9 (15.3–26.5)	17.9 (14.8–21.1)	18.2 (15.2–21.1)	10.6 (9.0–12.2) ***	−4.6 (−10.1 to +0.9)	0.10
Glycemic index	58.1 (56.2–59.9)	57.4 (55.3–59.5) *	57.7 (55.5–59.9)	54.4 (53.4–55.5) **	−2.6 (−5.7 to +0.5)	0.10
**Anthropometric variables**						
BMI (kg/m^2^)	33.6 (32.5–34.8)	33.4 (32.2–34.6)	33.1 (31.8–34.3)	31.2 (29.9–32.5) ***	−2.0 (−2.6 to −1.5)	<0.001
Lean mass (kg)	49.8 (46.2–53.4)	48.8 (45.4–52.2) **	50.6 (48.6–52.5)	48.3 (46.5–50.1) ***	−1.2 (−2.0 to −0.5)	0.002
Fat mass (kg)	39.1 (35.6–42.5)	39.5 (36.0–43.0)	42.0 (39.3–44.7)	38.1 (35.6–40.7) ***	−4.3 (−5.4 to −3.2)	<0.001
Visceral fat volume (cm^3^)	1434 (1154–1714)	1459 (1173–1744)	1289 (1040–1539)	1090 (864–1315) ***	−224 (−328 to −120)	<0.001
**Laboratory variables**						
Total cholesterol (mmol/L)	5.4 (5.0–5.8)	5.3 (5.0–5.6)	5.4 (5.1–5.7)	4.8 (4.4–5.2) ***	−1.1 (−2.0 to −0.2)	0.02
HDL-cholesterol (mmol/L)	1.6 (1.4–1.8)	1.6 (1.4–1.8)	1.6 (1.5–1.8)	1.4 (1.3–1.6) ***	−0.4 (−0.6 to −0.2)	0.002
LDL-cholesterol (mmol/L)	3.2 (2.9–3.6)	3.2 (2.9–3.5)	3.2 (2.9–3.5)	2.7 (2.3–3.1) **	−0.9 (−1.8 to −0.1)	0.03
Triglycerides (mmol/L)	1.1 (0.9–1.4)	1.2 (0.9–1.6)	1.2 (1.0–1.3)	1.4 (1.2–1.7) **	+0.9 (−0.3 to +2.2)	0.16
Fasting plasma glucose (mmol/L)	5.5 (5.3–5.7)	5.6 (5.4–5.8)	5.3 (5.1–5.6)	5.1 (4.9–5.2) **	−0.4 (−0.6 to −0.2)	<0.001
Fasting plasma insulin (pmol/L)	72.9 (56.9–88.2)	88.9 (38.9–135.4)	91.7 (72.9–111.8)	71.5 (55.6–87.5) **	−85.4 (−170.8 to +0.7)	0.05
Fasting plasma C-peptide (ng/mL)	2.5 (2.2–2.8)	3.0 (2.2–3.8)	2.6 (2.3–2.9)	2.1 (1.9–2.4) ***	−1.0 (−1.6 to −0.4)	0.003
HbA1c (DCCT, %)	5.8 (5.7–5.9)	5.8 (5.7–5.9)	5.8 (5.7–5.9)	5.8 (5.7–5.9)	0.0 (−0.1 to +0.1)	0.81
HbA1c (IFCC, mmol/mol)	40 (38.8–41.2)	40.1 (38.8–41.4)	39.9 (38.3–41.4)	40.1 (38.9–41.4)	+0.1 (−24.6 to +22.4)	0.81
**Insulin secretion/ Beta-cell function**						
Basal insulin secretion (pmol/min/m^2^)	100.5 (94.4–107.2)	104.1 (97.7–111.3)	108.6 (102.2–115.8)	83.0 (78.9–87.5) **	−54.2 (−86.5 to −21.9)	<0.001
Total insulin secretion (nmol/m^2^)	55.3 (52.6–58.2)	63.3 (60.4–66.4) **	53.8 (51.4–56.3)	54.8 (52.4–57.3)	−8.8 (−17.3 to +0.4)	0.07
Insulin secretion at a fixed glucose value (5 mM) (pmol/min/m^2^)	110.0 (99.6–121.1)	103.1 (93.9–112.8)	109.4 (100.1–119.4)	116.6 (105.9–128.1) *	+14.2 (−25.0 to +63.3)	0.10
Mean glucose (mmol/L)	5.5 (5.2–5.8)	6.1 (5.7–6.4) ***	5.6 (5.2–6.0)	5.4 (4.9–5.9) *	−0.8 (−1.2 to −0.4)	<0.001
Mean insulin (pmol/L)	312.2 (234.4–390.0)	406.4 (316.8–496.0) *	330.0 (259.4–400.6)	348.6 (274.7–422.5)	−75.7 (−180.4 to +29.1)	0.15
Glucose sensitivity (pmol/min/m^2^/mM)	107.5 (76.4–146.5)	181.1 (135.3–237.2)	108.5 (80.8–142.4)	213.8 (167.9–268.3) **	+65.5 (−74.4 to +205.4)	0.13
Rate sensitivity (pmol/m^2^/mM)	2260 (1419–3101)	1978 (1188–2768)	2783 (2127–3438)	2269 (1505–3033)	−232 (−1601 to +1137)	0.38
Potentiation factor ratio (dimensionless)	1.45 (1.28–1.63)	1.62 (1.43–1.82)	1.32 (1.17–1.47)	1.08 (0.95–1.22)	−0.51 (−1.04 to +0.03)	0.08
**Insulin sensitivity /resistance**						
3 h-oral glucose insulin sensitivity (mL/min/m^2^)	403.2 (389.1–417.6)	388.2 (374.5–402.3)	404.1 (391.7–416.8)	406.7 (394.2–419.5)	+10.8 (−29.5 to +51.1)	0.78
HOMA-IR (dimensionless)	2.4 (2.1–2.7)	2.8 (2.5–3.1)	2.5 (2.3–2.8)	1.9 (1.7–2.1) ***	−1.0 (−1.2 to −0.8)	0.004

## Abbreviations

BMI	body mass index
Gxt	the interaction between the factors group and time in the ANOVA model
HbA1c	glycated hemoglobin
HOMA-IR	Homeostasis Model Assessment Insulin Resistance
